# 1,3-Bis(1-cyclo­hexyl­eth­yl)imidazolidine-2-thione

**DOI:** 10.1107/S1600536812006150

**Published:** 2012-02-17

**Authors:** M. Naveed Umar, M. Nawaz Tahir, Mohammad Shoaib, Akbar Ali, Ziauddin

**Affiliations:** a Department of Chemistry, University of Malakand, Pakistan; b University of Sargodha, Department of Physics, Sargodha, Pakistan; c Department of Pharmacy, University of Malakand, Pakistan

## Abstract

The complete mol­ecule of the title compound, C_19_H_34_N_2_S, is generated by crystallographic twofold symmetry, with the C=S group lying on the rotation axis. A short C—H⋯S contact occurs in the mol­ecule. The five-membered ring is twisted and the cyclo­hexyl ring adopts a chair conformation. The dihedral angle between the mean plane of the five-membered ring and the basal plane of the cyclo­hexyl ring is 75.32 (13)°.

## Related literature

For a related structure, see: Kazak *et al.* (2005 ▶).
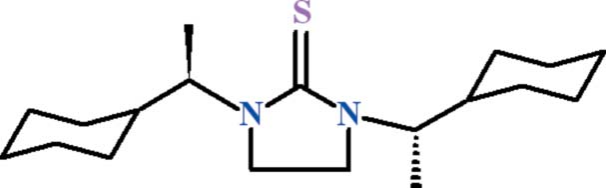


## Experimental

### 

#### Crystal data

C_19_H_34_N_2_S
*M* *_r_* = 322.54
Tetragonal, 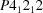

*a* = 6.1008 (3) Å
*c* = 53.790 (2) Å
*V* = 2002.04 (17) Å^3^
*Z* = 4
Mo *K*α radiation
μ = 0.16 mm^−1^
*T* = 296 K
0.30 × 0.25 × 0.20 mm

#### Data collection

Bruker Kappa APEXII CCD diffractometer
Absorption correction: multi-scan (*SADABS*; Bruker, 2005 ▶) *T*_min_ = 0.957, *T*_max_ = 0.966
18805 measured reflections
2500 independent reflections
1357 reflections with *I* > 2σ(*I*)
*R*_int_ = 0.047

#### Refinement

*R*[*F* ^2^ > 2σ(*F* ^2^)] = 0.060
*wR*(*F* ^2^) = 0.176
*S* = 1.04
2500 reflections
102 parameters
H-atom parameters constrained
Δρ_max_ = 0.12 e Å^−3^
Δρ_min_ = −0.12 e Å^−3^
Absolute structure: Flack (1983 ▶), with 874 Friedel pairs
Flack parameter: 0.0 (2)

### 

Data collection: *APEX2* (Bruker, 2007 ▶); cell refinement: *SAINT* (Bruker, 2007 ▶); data reduction: *SAINT*; program(s) used to solve structure: *SHELXS97* (Sheldrick, 2008 ▶); program(s) used to refine structure: *SHELXL97* (Sheldrick, 2008 ▶); molecular graphics: *ORTEP-3 for Windows* (Farrugia, 1997 ▶) and *PLATON* (Spek, 2009 ▶); software used to prepare material for publication: *WinGX* (Farrugia, 1999 ▶) and *PLATON*.

## Supplementary Material

Crystal structure: contains datablock(s) global, I. DOI: 10.1107/S1600536812006150/hb6637sup1.cif

Structure factors: contains datablock(s) I. DOI: 10.1107/S1600536812006150/hb6637Isup2.hkl

Supplementary material file. DOI: 10.1107/S1600536812006150/hb6637Isup3.cml

Additional supplementary materials:  crystallographic information; 3D view; checkCIF report

## Figures and Tables

**Table 1:** Hydrogen-bond geometry (Å, °)

*D*—H⋯*A*	*D*—H	H⋯*A*	*D*⋯*A*	*D*—H⋯*A*
C3—H3⋯S1	0.98	2.65	3.174 (3)	114

**Fig. 1.**
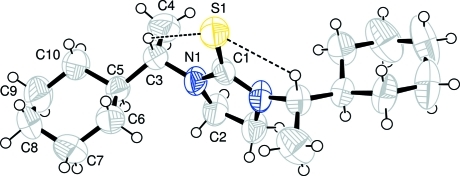
View of the title compound with displacement ellipsoids drawn at the 50% probability level. The dotted lines indicate the short C—H···S contacts.

## supplementary crystallographic information

### Comment

The title compound (I), (Fig. 1) has been synthesized as a part of our project related to imidazolidinethione.

The crystal structure of 1,3-dibenzoyl-4,5-dihydro-1*H*-imidazole-2(3*H*)-thione (Kazak *et al.*, 2005) has been published which is related to the title compound (I), (Fig. 1).

The molecule has twofold symmetry about the C═S (C1═S1) of imidazolidinethione and therefore, the asymmetric unit is half of the molecule. The asymmetric part of imidazolidinethione moiety A (S1/C1/N1/C2) and the basal plane of cyclohexyl ring B (C6/C7/C9/C10) are almost planar with r.m.s. deviations of 0.036 and 0.004 Å, respectively. The dihedral angle between A/B is 75.32 (13)°. The cyclohexyl adopts chair conformation with apical C-atoms C5 and C8 at a distance of -0.651 (5) and 0.638 (8) Å, respectively from the basal plane B. There exist weak intramolecular H-bondings of C—H···S type (Table 1, Fig. 1) and form S(5) ring motif. No other interaction is found in the crystal.

### Experimental

(*S*)-1-cyclohexylethanamine (2.5 equiv.) and 1,2-dibromoethane (1 equiv.) were placed in a pressure vessel and heated at 393 K for 5 h, during which the reaction mixture solidified. The system was cooled to room temperature and NaOH (1 N, 20 ml) and ethyl acetate (20 ml) were added in to the reaction mixture. After dissolving the reaction mixture, the crude product was extracted with ethyl acetate (3 × 25 ml). The combined organic layers were concentrated and subjected to column chromatography. The product obtained from column chromatography (1 equiv.) was added to toluene (0.4 *M*) in pressure vessel and thiocarbonyldiimidazol (1.1 equiv.) was added to it. This mixture was heated about 373 K for 15 h. Again the extraction with ethyl acetate (3 × 25 ml) was carried out by using column chromatography to get the required product. Yield: 90%. Colourless prisms of (I) were obtained by recrystallizing from methanol after 48 h.

### Refinement

The H atoms were positioned geometrically (C—H = 0.93–0.98 Å) and refined as riding with *U*_iso_(H) = x*U*_eq_(C), where x = 1.5 for methyl and x = 1.2 for all other H atoms.

### Crystal data

**Table:**

C_19_H_34_N_2_S	*D*_x_ = 1.070 Mg m^−^^3^
*M**_r_* = 322.54	Mo *K*α radiation, λ = 0.71073 Å
Tetragonal, *P*4_1_2_1_2	Cell parameters from 1358 reflections
Hall symbol: P 4abw 2nw	θ = 3.0–28.3°
*a* = 6.1008 (3) Å	µ = 0.16 mm^−^^1^
*c* = 53.790 (2) Å	*T* = 296 K
*V* = 2002.04 (17) Å^3^	Prism, colourless
*Z* = 4	0.30 × 0.25 × 0.20 mm
*F*(000) = 712	

### Data collection

**Table:**

Bruker Kappa APEXII CCD diffractometer	2500 independent reflections
Radiation source: fine-focus sealed tube	1357 reflections with *I* > 2σ(*I*)
Graphite monochromator	*R*_int_ = 0.047
Detector resolution: 7.50 pixels mm^-1^	θ_max_ = 28.3°, θ_min_ = 3.0°
ω scans	*h* = −8→4
Absorption correction: multi-scan (*SADABS*; Bruker, 2005)	*k* = −7→8
*T*_min_ = 0.957, *T*_max_ = 0.966	*l* = −71→65
18805 measured reflections	

### Refinement

**Table:**

Refinement on *F*^2^	Secondary atom site location: difference Fourier map
Least-squares matrix: full	Hydrogen site location: inferred from neighbouring sites
*R*[*F*^2^ > 2σ(*F*^2^)] = 0.060	H-atom parameters constrained
*wR*(*F*^2^) = 0.176	*w* = 1/[σ^2^(*F*_o_^2^) + (0.069*P*)^2^ + 0.4327*P*] where *P* = (*F*_o_^2^ + 2*F*_c_^2^)/3
*S* = 1.04	(Δ/σ)_max_ < 0.001
2500 reflections	Δρ_max_ = 0.12 e Å^−^^3^
102 parameters	Δρ_min_ = −0.12 e Å^−^^3^
0 restraints	Absolute structure: Flack (1983), with 874 Friedel pairs
Primary atom site location: structure-invariant direct methods	Flack parameter: 0.0 (2)

### Special details

**Table:**

Geometry. Bond distances, angles *etc*. have been calculated using the rounded fractional coordinates. All su's are estimated from the variances of the (full) variance-covariance matrix. The cell e.s.d.'s are taken into account in the estimation of distances, angles and torsion angles
Refinement. Refinement of *F*^2^ against ALL reflections. The weighted *R*-factor *wR* and goodness of fit *S* are based on *F*^2^, conventional *R*-factors *R* are based on *F*, with *F* set to zero for negative *F*^2^. The threshold expression of *F*^2^ > σ(*F*^2^) is used only for calculating *R*-factors(gt) *etc*. and is not relevant to the choice of reflections for refinement. *R*-factors based on *F*^2^ are statistically about twice as large as those based on *F*, and *R*-factors based on ALL data will be even larger.

### Fractional atomic coordinates and isotropic or equivalent isotropic displacement parameters (Å^2^)

**Table:**

	*x*	*y*	*z*	*U*_iso_*/*U*_eq_	
S1	0.56724 (12)	0.56724 (12)	0.0000	0.0822 (4)	
N1	0.2683 (4)	0.2943 (4)	0.02021 (4)	0.0735 (7)	
C1	0.3729 (4)	0.3729 (4)	0.0000	0.0652 (10)	
C2	0.0922 (6)	0.1442 (6)	0.01337 (5)	0.0868 (10)	
H2A	−0.0500	0.2138	0.0149	0.104*	
H2B	0.0950	0.0131	0.0236	0.104*	
C3	0.2873 (5)	0.3852 (5)	0.04524 (5)	0.0696 (8)	
H3	0.4132	0.4849	0.0451	0.083*	
C4	0.0864 (7)	0.5222 (6)	0.05128 (8)	0.1131 (13)	
H4A	0.1147	0.6093	0.0658	0.170*	
H4B	−0.0361	0.4273	0.0544	0.170*	
H4C	0.0537	0.6167	0.0375	0.170*	
C5	0.3390 (5)	0.2040 (5)	0.06392 (4)	0.0652 (8)	
H5	0.2139	0.1032	0.0643	0.078*	
C6	0.5406 (6)	0.0737 (6)	0.05665 (6)	0.0915 (10)	
H6A	0.5139	0.0013	0.0409	0.110*	
H6B	0.6630	0.1732	0.0544	0.110*	
C7	0.6000 (9)	−0.0961 (7)	0.07594 (8)	0.1415 (18)	
H7A	0.4867	−0.2075	0.0765	0.170*	
H7B	0.7363	−0.1666	0.0712	0.170*	
C8	0.6254 (10)	0.0031 (7)	0.10136 (8)	0.147 (2)	
H8A	0.6529	−0.1123	0.1134	0.177*	
H8B	0.7506	0.1011	0.1014	0.177*	
C9	0.4288 (10)	0.1250 (8)	0.10869 (7)	0.1370 (18)	
H9A	0.4537	0.1940	0.1247	0.164*	
H9B	0.3070	0.0240	0.1105	0.164*	
C10	0.3709 (7)	0.2961 (6)	0.09005 (5)	0.0976 (12)	
H10A	0.4863	0.4054	0.0896	0.117*	
H10B	0.2369	0.3685	0.0952	0.117*	

### Atomic displacement parameters (Å^2^)

**Table:**

	*U*^11^	*U*^22^	*U*^33^	*U*^12^	*U*^13^	*U*^23^
S1	0.0833 (6)	0.0833 (6)	0.0798 (7)	−0.0262 (6)	−0.0097 (5)	0.0097 (5)
N1	0.0914 (18)	0.0764 (16)	0.0526 (12)	−0.0234 (13)	−0.0101 (12)	0.0110 (12)
C1	0.0661 (15)	0.0661 (15)	0.063 (2)	−0.0067 (19)	−0.0152 (14)	0.0152 (14)
C2	0.095 (2)	0.099 (3)	0.0666 (16)	−0.037 (2)	−0.0083 (16)	0.0109 (16)
C3	0.086 (2)	0.0616 (18)	0.0614 (16)	−0.0014 (16)	−0.0048 (15)	0.0017 (13)
C4	0.121 (3)	0.091 (3)	0.128 (3)	0.026 (3)	−0.024 (3)	−0.017 (2)
C5	0.081 (2)	0.0624 (16)	0.0520 (14)	−0.0071 (15)	0.0022 (14)	0.0016 (13)
C6	0.109 (3)	0.087 (2)	0.079 (2)	0.027 (2)	−0.0062 (19)	−0.0082 (18)
C7	0.181 (5)	0.087 (3)	0.157 (4)	0.048 (3)	−0.061 (4)	−0.011 (3)
C8	0.238 (7)	0.094 (3)	0.110 (3)	0.014 (4)	−0.088 (4)	0.021 (2)
C9	0.219 (6)	0.124 (4)	0.068 (2)	−0.022 (4)	−0.019 (3)	0.024 (2)
C10	0.144 (4)	0.094 (2)	0.0548 (16)	0.008 (2)	0.0080 (19)	−0.0014 (17)

### Geometric parameters (Å, º)

**Table:**

S1—C1	1.677 (3)	C3—H3	0.9800
N1—C1	1.349 (3)	C4—H4A	0.9600
N1—C2	1.458 (4)	C4—H4B	0.9600
N1—C3	1.461 (4)	C4—H4C	0.9600
C2—C2^i^	1.506 (4)	C5—H5	0.9800
C3—C4	1.519 (5)	C6—H6A	0.9700
C3—C5	1.527 (4)	C6—H6B	0.9700
C5—C6	1.516 (5)	C7—H7A	0.9700
C5—C10	1.526 (4)	C7—H7B	0.9700
C6—C7	1.510 (6)	C8—H8A	0.9700
C7—C8	1.503 (6)	C8—H8B	0.9700
C8—C9	1.465 (8)	C9—H9A	0.9700
C9—C10	1.490 (6)	C9—H9B	0.9700
C2—H2A	0.9700	C10—H10A	0.9700
C2—H2B	0.9700	C10—H10B	0.9700
			
C1—N1—C2	111.6 (2)	H4A—C4—H4C	109.00
C1—N1—C3	124.8 (2)	H4B—C4—H4C	109.00
C2—N1—C3	122.0 (2)	C3—C5—H5	108.00
S1—C1—N1	125.87 (13)	C6—C5—H5	108.00
S1—C1—N1^i^	125.87 (13)	C10—C5—H5	108.00
N1—C1—N1^i^	108.3 (2)	C5—C6—H6A	109.00
N1—C2—C2^i^	102.6 (3)	C5—C6—H6B	109.00
N1—C3—C4	110.0 (3)	C7—C6—H6A	109.00
N1—C3—C5	110.4 (2)	C7—C6—H6B	109.00
C4—C3—C5	115.1 (3)	H6A—C6—H6B	108.00
C3—C5—C6	112.2 (2)	C6—C7—H7A	109.00
C3—C5—C10	111.5 (3)	C6—C7—H7B	109.00
C6—C5—C10	109.1 (3)	C8—C7—H7A	109.00
C5—C6—C7	112.2 (3)	C8—C7—H7B	109.00
C6—C7—C8	111.9 (3)	H7A—C7—H7B	108.00
C7—C8—C9	111.4 (4)	C7—C8—H8A	109.00
C8—C9—C10	111.6 (4)	C7—C8—H8B	109.00
C5—C10—C9	113.1 (3)	C9—C8—H8A	109.00
N1—C2—H2A	111.00	C9—C8—H8B	109.00
N1—C2—H2B	111.00	H8A—C8—H8B	108.00
H2A—C2—H2B	109.00	C8—C9—H9A	109.00
C2^i^—C2—H2A	111.00	C8—C9—H9B	109.00
C2^i^—C2—H2B	111.00	C10—C9—H9A	109.00
N1—C3—H3	107.00	C10—C9—H9B	109.00
C4—C3—H3	107.00	H9A—C9—H9B	108.00
C5—C3—H3	107.00	C5—C10—H10A	109.00
C3—C4—H4A	109.00	C5—C10—H10B	109.00
C3—C4—H4B	109.00	C9—C10—H10A	109.00
C3—C4—H4C	109.00	C9—C10—H10B	109.00
H4A—C4—H4B	109.00	H10A—C10—H10B	108.00
			
C2—N1—C1—S1	173.7 (2)	N1—C3—C5—C10	177.2 (3)
C2—N1—C1—N1^i^	−6.3 (3)	C4—C3—C5—C6	179.8 (3)
C3—N1—C1—S1	8.2 (4)	C4—C3—C5—C10	−57.6 (4)
C3—N1—C1—N1^i^	−171.8 (2)	C3—C5—C6—C7	176.4 (3)
C1—N1—C2—C2^i^	15.4 (3)	C10—C5—C6—C7	52.4 (4)
C3—N1—C2—C2^i^	−178.6 (3)	C3—C5—C10—C9	−178.1 (4)
C1—N1—C3—C4	102.2 (3)	C6—C5—C10—C9	−53.7 (4)
C1—N1—C3—C5	−129.7 (3)	C5—C6—C7—C8	−54.2 (5)
C2—N1—C3—C4	−61.9 (4)	C6—C7—C8—C9	55.0 (5)
C2—N1—C3—C5	66.2 (3)	C7—C8—C9—C10	−55.6 (5)
N1—C2—C2^i^—N1^i^	−17.5 (3)	C8—C9—C10—C5	56.3 (5)
N1—C3—C5—C6	54.5 (3)		

Symmetry code: (i) *y*, *x*, −*z*.

### Hydrogen-bond geometry (Å, º)

**Table:**

*D*—H···*A*	*D*—H	H···*A*	*D*···*A*	*D*—H···*A*
C3—H3···S1	0.98	2.65	3.174 (3)	114
